# miR-221 Augments TRAIL-Mediated Apoptosis in Prostate Cancer Cells by Inducing Endogenous TRAIL Expression and Targeting the Functional Repressors SOCS3 and PIK3R1

**DOI:** 10.1155/2019/6392748

**Published:** 2019-11-14

**Authors:** Markus Krebs, Christoph Behrmann, Charis Kalogirou, Ioannis Sokolakis, Susanne Kneitz, Marianna Kruithof-de Julio, Eugenio Zoni, Anne Rech, Bastian Schilling, Hubert Kübler, Martin Spahn, Burkhard Kneitz

**Affiliations:** ^1^Department of Urology and Paediatric Urology, University Hospital Würzburg, Würzburg, Germany; ^2^Else Kröner Integrative Clinician Scientist College for Translational Immunology, University Hospital Würzburg, Würzburg, Germany; ^3^Physiological Chemistry, University of Würzburg, Biocentre, Würzburg, Germany; ^4^Urology Research Laboratory, Department of Biomedical Research, University of Bern, Bern, Switzerland; ^5^Department of Dermatology, Venereology and Allergology, University Hospital Würzburg, Würzburg, Germany; ^6^Urology Hirslanden Zürich, Zürich, Switzerland

## Abstract

miR-221 is regarded as an oncogene in many malignancies, and miR-221-mediated resistance towards TRAIL was one of the first oncogenic roles shown for this small noncoding RNA. In contrast, miR-221 is downregulated in prostate cancer (PCa), thereby implying a tumour suppressive function. By using proliferation and apoptosis assays, we show a novel feature of miR-221 in PCa cells: instead of inducing TRAIL resistance, miR-221 sensitized cells towards TRAIL-induced proliferation inhibition and apoptosis induction. Partially responsible for this effect was the interferon-mediated gene signature, which among other things contained an endogenous overexpression of the TRAIL encoding gene TNFSF10. This TRAIL-friendly environment was provoked by downregulation of the established miR-221 target gene SOCS3. Moreover, we introduced PIK3R1 as a target gene of miR-221 in PCa cells. Proliferation assays showed that siRNA-mediated downregulation of SOCS3 and PIK3R1 mimicked the effect of miR-221 on TRAIL sensitivity. Finally, Western blotting experiments confirmed lower amounts of phospho-Akt after siRNA-mediated downregulation of PIK3R1 in PC3 cells. Our results further support the tumour suppressing role of miR-221 in PCa, since it sensitises PCa cells towards TRAIL by regulating the expression of the oncogenes SOCS3 and PIK3R1. Given the TRAIL-inhibiting effect of miR-221 in various cancer entities, our results suggest that the influence of miR-221 on TRAIL-mediated apoptosis is highly context- and entity-dependent.

## 1. Introduction

Tumour Necrosis Factor Related Apoptosis Inducing Ligand (TRAIL) is a promising target in cancer therapy, since activation of TRAIL receptors (also called death receptors) located specifically at the surface of tumour cells induces apoptosis, whereas surrounding benign tissue stays unaffected [1]. This potential has led to a plethora of TRAIL-based cancer therapies currently being tested in (pre-)clinical studies [2]. However, evolving resistance of cancer cells towards TRAIL is a serious limitation for these therapeutic strategies. To overcome resistance, combining TRAIL with other compounds like cisplatin or Tyrosine Kinase inhibitors (TKI) has been evaluated [3, 4]. In this context, the influence of microRNAs (miRs) on TRAIL-mediated apoptosis has been studied in several cancer entities [5]. miRs are RNA strands consisting of 20–25 nucleotides, which negatively regulate gene expression of hundreds of target genes by binding to their corresponding mRNA strand, thereby stopping further translation.

One miR candidate well known for inhibiting TRAIL effects in cancer cells is miR-221. This feature has been shown in hepatocellular carcinoma (HCC), non-small cell lung cancer (NSCLC) and bladder cancer cells [6, 7] and seems to be in line with publications claiming an oncogenic role for miR-221 in many malignancies [8]. In contrast, studies by others and our group [9, 10] were able to show a significant downregulation of miR-221 in PCa tissue, thus suggesting a role as a tumour suppressor and a potential biomarker predicting overall and cancer-specific survival of PCa patients. We also demonstrated that a restoration of cellular miR-221 expression levels in PCa cells induced an interferon-mediated gene signature [11]. This effect was at least partly caused by miR-221 targeting IRF2 and SOCS3, two repressors of JAK-STAT-mediated pathways. As TRAIL and interferon signalling frequently act concordantly and TRAIL itself belongs to the group of interferon-induced genes [12, 13], we wanted to investigate the influence of miR-221 on TRAIL effects in PCa and to evaluate the role of miR-221-mediated regulation of TRAIL signalling regarding the tumour suppressive function of miR-221.

## 2. Materials and Methods

### 2.1. Cell Culture and Chemicals

We obtained the human cancer cell lines PC3, DU145, LNCaP, and RWPE cells from American Tissue Collection Center (ATCC) and cultured them according to the recommended protocols. All media were supplemented with 10% fetal calf serum, and 1% penicillin/streptomycin. Unless stated otherwise, TRAIL (PeproTech) was administered 48 h after plating cells in a final concentration of 10 ng/ml.

### 2.2. Proliferation Assays/MTS Assays and Transfection

Proliferation of PC3, DU145, LNCaP, and RWPE cells was examined in triplicates in 96-well plates. Transient transfections of pre-miR-221 or siRNA SOCS3 with the respective controls were carried out as published previously [11]. The following short interfering RNA sequence was used for targeting human PIK3R1: 5′-CCCAGUGUAGCAUCCUAAATT-3′ obtained from Qiagen (FlexiTube siRNA). Efficient downregulation of PIK3R1 in PIK3R1 siRNA-transfected cells was confirmed by qRT-PCR and Western blotting. Scrambled, nontargeting control-siRNA or control-pre-miRNA were purchased from Qiagen. Cells were transfected either with human precursor miR-221 (pre-miR-221, 50 nmol/l, Ambion), siRNA (50 nmol/l, Qiagen), or respective controls using the Lipofectamine 2000 reagent (Invitrogen) 24 h after plating. 48 h and/or 120 h after transient transfection and TRAIL treatment, cells were examined with MTS Cell Titer 96 Proliferation Assay (Promega) at 490 nm with a monochromator (Biorad). All experiments were analysed as triplicates. Each result consisted of at least five independent experiments.

### 2.3. Apoptosis Assays

We analysed Caspase-3/7 activity using the Caspase-GLO 3/7 Kit (Promega) as previously described [11]. Cells were transfected with pre-miR-221, siRNAs, and corresponding ctr-RNAs as described above. After indicated timepoints, cells were incubated with medium supplemented with Caspase-3/7 reagent for 4 h at room temperature. Cells were lysated as recommended by the manufacturer's instructions and transferred to a white-walled 96-well plate for measurement of luminescence. Data were expressed as OD values and normalised to the control-transfected cells. Experiments were performed as triplicates. Each result consisted of five independent experiments.

### 2.4. RNA Extraction, Reverse Transcription and qRT-PCR

Total RNA was extracted from the cells using TRIzol reagent (Life Technologies). The RNA concentration and integrity was determined with a bioanalyzer (Agilent). Reverse transcriptions were performed as described previously [11]. mRNA expression analyses of XAF1, TNFSF10, STAT1, SOCS3, and PIK3R1 were performed according to standard qRT-PCR procedures. All primer sequences are available upon request. Primers were obtained from Qiagen and analysed by the QuantiTect primer system as recommended by the manufacturer. Mean *C*_*t*_ was determined from duplicate PCR runs. Values showing a standard deviation higher than 0.5 were excluded from further analysis. The expression of *β*-Actin was used for normalisation. The 2^–∆∆Ct^ method was used to assess fold changes in mRNA expression between samples and controls. Each result consisted of at least five independent experiments.

### 2.5. Western Blotting Experiments

Harvested cells were washed with PBS and lysed in PhosphoSafe (Novagen) following the instructions of the manufacturer. Total protein concentrations were quantified (Bradford reagent) and protein isolates were loaded on 12.5% SDS-PAGE gels and later transferred to nitrocellulose membranes (Bio-Rad). The membranes were blocked using block-buffer (Invitrogen) and incubated at 4°C with the primary antibody. We used the following antibodies: PIK3R1 (N-term L11, anti-Rabbit, Sigma Aldrich), Akt 1/2/3 (5C10, anti-Mouse, Santa Cruz), p-Akt (D9E, anti-Rabbit, Cell Signaling), STAT3 (EPR787Y, anti-Rabbit, Abcam), p-STAT3 (EP2147Y, anti-Rabbit, Abcam), cleaved PARP1 (E51, anti-Rabbit, Abcam). ERK-2 (C14, anti-Rabbit, Santa Cruz) was used as loading control. We utilised horseradish peroxidase–coupled secondary antibodies and the ECL Plus system (GE Healthcare) to visualize the protein expression. All experiments were repeated at least three times.

### 2.6. Luciferase Reporter Assays

Cloning procedure and plasmid use were described previously [11]. Human *PIK3R1* 3′-UTR construct containing the putative miR-221 binding site was PCR-amplified using the following primers that contained additional Hind III I sites: piK3r1Hindfor: 5′-GATCAAGCTTTCTGAAGCTTTACCAGCTG-3′; pik3r1Hind rev: 5′-CAAT AAGCTTGTGGG GAAGCTTATTCTG-3′. The resulting *PIK3R1 *PCR fragment containing the miR-221 complementary site to *pik3r1* was cloned into a luciferase reporter plasmid (pMIR- REPORT-Luciferase, Applied Biosystems) downstream of the Renilla luciferase stop codon using Hind III sites. The resulting reporter vector (50 ng/well) was co-transfected with a control nontargeting RNA oligonucleotide (ctrl) or pre-miR-221 (50 nmol/l). Lipofectamine™ reagent (Invitrogen) was used for transient transfection. To perform luciferase assays, LNCaP cells or PC3 cells were plated in 6-well plates (3.5 × 10^5^ or 2 × 10^5^ cells per well) and incubated for 24 h before transfection. 48 h after transfection, the Firefly- and Renilla-Luciferase activity was analysed using the Dual-Luciferase® Reporter Assay System (Promega) as recommended by the manufacturer's protocols. Renilla activity was used for normalisation and as a control for transfection efficiency in each individual analysis. Using the Site-Directed Mutagenesis Kit (QuickChange, Agilent Technologies), we introduced a mutation into the miR-221 binding site of *PIK3R1*. An additional Mlu restriction site was introduced for screening. Primers for mutagenesis are as follows: pik3r1Mutfor: 5′-TGACTCGTTCATTTGTTACAAAATGAAATAATC3′, pik3r1Mutrev: 5′-CGCGTGAAAGGCACGTCCACTCA3′. The resulting pik3r1Mut reporter vector was used to confirm specific binding of miR-221 at the putative binding site. All cloning steps were verified by sequencing of resulting constructs. All reporter assays transfected with the mutant or wildtype *PIK3R1*-3′UTR were repeated at least four times.

### 2.7. Statistical and Bioinformatical Analyses

Student's unpaired *t*-tests were applied to discriminate significant differences in two groups of normally distributed data. For multiple groups, we applied ANOVA with subsequent Bonferroni post hoc testing (correcting for multiple tests). Significance levels were determined as *α* = 95% and *α* = 99%. Statistically significant associations were set as ∗*p* < 0.05 and ∗∗*p* < 0.01, respectively. We used R build 3.2.2 (https://www.r-project.org/) for statistical evaluation.

For bioinformatical analysis of TCGA data, we utilised the freely accessible *UALCAN* web resource for determining genes co-expressed with TNFSF10/TRAIL in PCa tissue [14]. The TNFSF10/TRAIL-associated signature (top 50 co-expressed genes in PCa) was further examined via *Reactome Pathway *analysis [15, 16]. New functional annotation analyses of expression data from microarray experiments earlier performed by our group [11] were conducted using *DAVID Functional Annotation* [17, 18]. *Targetscan.com* was used as a tool for predicting miR-mRNA targeting, specifically to detect the miR-221 binding sequence at the 3′UTR of PIK3R1 [19].

## 3. Results

### 3.1. miR-221 Sensitised PCa Cells towards TRAIL-Mediated Effects on Cell Viability and Apoptosis Induction

**Figure 1:**
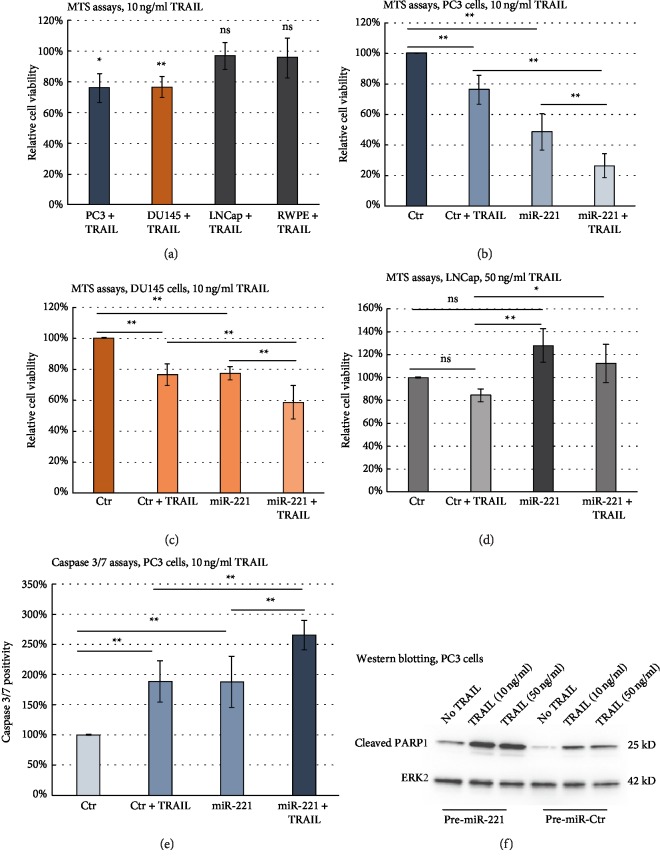
Overexpression of miR-221 augmented TRAIL effects in terms of cell viability and apoptosis induction in PCa cells. (a) TRAIL administration (final concentration of 10 ng/ml) led to a significantly lower proportion of proliferating PC3 and DU145 cells. In contrast, LNCaP cells and nonmalignant RWPE cells did not show a significant change in viability after TRAIL administration. Relative cell viability was normalised to the viability of untreated control cell lines, which was arbitrarily set as 100% (b, c). Transient transfection with pre-miR-221 significantly augmented TRAIL sensitivity in terms of cell viability in (b) PC3 and (c) DU145 cells (120 h p. t.). (d) Higher doses of TRAIL (50 ng/ml) led to a decrease in cell viability of LNCaP cells. Overexpression of miR-221 caused increased cell viability. In combination, pre-miR-221 transfected LNCaP cells were significantly less susceptible towards TRAIL effects compared to control transfections. (b, c, d) Relative cell viability was normalised to the viability of control cells, which was arbitrarily set as 100%. Control cells (Ctr) were transfected with a scrambled, non targeting pre-miRNA. (e) miR-221 overexpression significantly augmented Caspase 3/7 activity in PC3 cells. 10 ng/ml TRAIL was administered 24 h p. t. Experiments took place 48 h p. t. Caspase positivity was normalized to the Caspase activity of control cells, which was arbitrarily set as 100%. Control cells (Ctr) were transfected with a scrambled, non targeting pre-miRNA . (a, b, c, d, e) Data represent mean + SD from five independent experiments, ∗*p* < 0.05; ∗∗*p* < 0.01. (b, c, d, e) ANOVA with Bonferroni post hoc testing. (f) Western blotting experiments confirmed higher expression levels of cleaved PARP1 in pre-miR-221 transfected PC3 cells compared to pre-miR-control transfections (48 h p. t.). TRAIL treatment in doses of 10 ng/ml and 50 ng/ml caused higher expression levels of cleaved PARP1 in pre-miR-Control and pre-miR-221 transfected PC3 cells compared to untreated cells–with higher levels in TRAIL-treated cells overexpressing miR-221.

Figure 1(a) illustrates the effects of TRAIL administration (10 ng/ml) on survival of PC3, DU145 and LNCaP cells as well as RWPE cells. Whereas TRAIL caused a significant reduction of cell viability in PC3 (76.1 ± 9.4% cells compared to control, *p* = 0.011) and DU145 cells (76.6 ± 6.8% viable cells, *p* = 0.006), no significant effect of 10 ng/ml TRAIL administration could be detected in LNCaP cells (96.8 ± 8.8%, *p* = 0.52) or in nonmalignant RWPE cells (95.8 ± 12.9%, *p*=0.64).

We also looked at potential additional effects of miR-221 overexpression on TRAIL-treated PC3 and DU145 cells (Figures 1(b) and 1(c)). Compared to control transfected cells, TRAIL administration after transfection with pre-miR-221 caused a significant further reduction in cell viability of PC3 (26.5 ± 7.9% in comparison to 76.1 ± 9.4% viable cells, *p* < 0.01) and DU145 cells (58.6 ± 10.9% compared to 76.6 ± 6.8% viable cells, *p* < 0.01). As shown before, pre-mir-221 transfection in comparison to control transfections led to a significant reduction in cell viability of PC3 (48.6 ± 12.1%, *p* < 0.01) and DU145 cells (77.5 ± 4.4%, *p* < 0.01). Although higher doses of TRAIL (50 ng/ml) led to a decrease in cell viability of LNCaP cells (Figure 1(d)), this decline did not meet statistical significance criteria. Whereas miR-221 overexpressing LNCaP cells showed a trend towards higher cell viability (*p* = 0.054), the comparison between TRAIL-treated LNCaP cells after pre-miR-221 or control transfections was statistically significant (119.5 ± 19.7% vs. 84.8 ± 5.6%, *p* = 0.013). As shown for PC3 cells by Caspase 3/7 assays (Figure 1(e)), TRAIL administration compared to control strengthened relative apoptosis in a highly significant manner (188.6 ± 34.4%, *p* < 0.01). Comparable results could be seen after pre-miR-221 transfections (187.8 ± 42.9%, *p* < 0.01). Co-treatment with pre-miR-221 transfection and TRAIL administration resulted in a highly significant additional effect on apoptosis induction (266 ± 24.8%, *p* < 0.01). To confirm this increased apoptotic death rate observed in TRAIL-treated and miR-221 overexpressing PC3 cells, we additionally analysed the expression of cleaved PARP1 (Figure 1(f)). We found an increased expression of cleaved PARP1 in TRAIL-treated, control-transfected cells and a strengthened expression of cleaved PARP1 in miR-221 overexpressing cells, indicating that miR-221 increased the sensitivity of PC3 cells towards TRAIL-induced apoptosis.

### 3.2. miR-221 Induced Endogenous TRAIL Expression and a TRAIL-Friendly Milieu of Interferon-Related Genes

**Figure 2:**
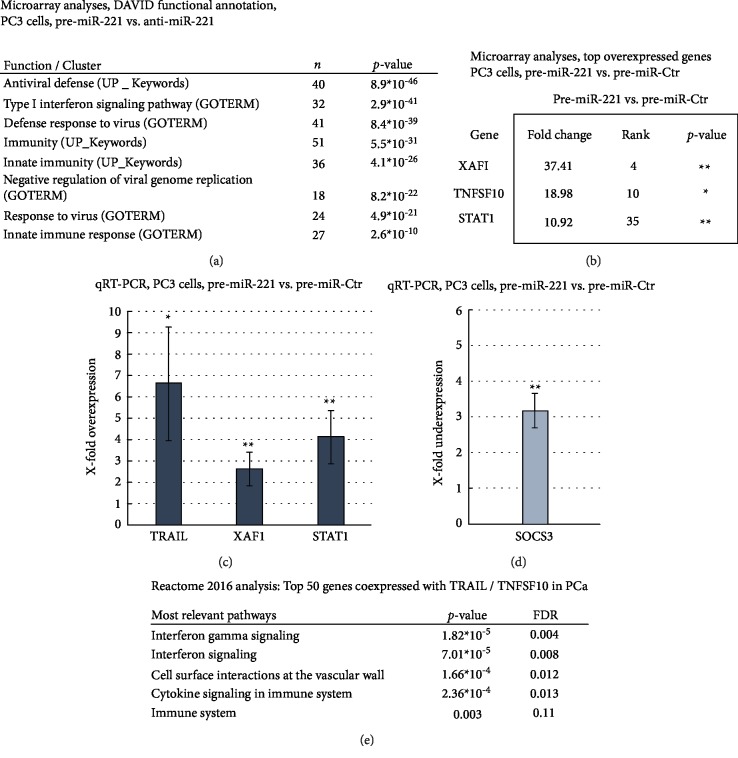
Interaction of TRAIL and interferon signalling in PCa tissue and cells and its modulation by miR-221 expression. (a) *DAVID Functional Annotation* analysis of Affymetrix microarray data (PC3 cells, pre-miR-221 vs. anti-miR-221 transfections, 48 h p. t.) identified interferon-related cellular functions as being significantly enriched. (b) Significant overexpression of XAF1, TNFSF10, and STAT1 in miR-221 overexpressing PC3 cells within microarray experiments (pre-miR-221 vs- anti-miR-221 and pre-miR-221 vs. pre-miR-Ctr, 48 h p. t.). (c) qRT-PCR confirmed a significant upregulation of TNFSF10, XAF1, and STAT1 expression as well as (d) a significant downregulation of SOCS3 after restoration of miR-221 levels in PC3 cells compared to control (48 h p. t.). Data represent mean + SD from five independent experiments, ∗*p* < 0.05; ∗∗*p* < 0.01. (e) *Reactome pathway* analysis of top 50 genes positively correlated with TNFSF10/TRAIL expression within TCGA dataset (Prostate Adenocarcinoma, PanCancer Atlas). FDR: false discovery rate.

Based on the observed sensitisation of miR-221 overexpressing PC3 and DU145 cells towards TRAIL-induced cell death, we were interested in understanding the molecular changes induced by miR-221. As illustrated in Figure 2(a), we performed a *DAVID Functional Annotation* analysis using gene expression signatures of pre-miR-221 transfected PC3 cells (48 h p. t.) detected by microarray experiments. We identified immune and interferon-related pathways as significantly enriched in PC3 cells with high miR-221 expression levels. Among the top ranked genes within these microarray analyses, we saw XAF1, TNFSF10, and STAT1 (Figure 2(b)). Additionally, we confirmed these results by qRT-PCR experiments (Figures 2(c) and 2(d)) showing significant overexpression for XAF1, the TRAIL encoding gene TNFSF10 and STAT1 along with a downregulation of SOCS3 in miR-221 overexpressing PCa cells. Since we know that miR-221 overexpression is linked to a signature of interferon-induced genes, we examined whether TRAIL signalling is linked to interferon signalling also in PCa specimen. Therefore, we determined the top 50 genes co-expressed with TNFSF10 in PCa tissue (TCGA dataset, Prostate Adenocarcinoma, PanCancer Atlas). Further *Reactome pathway* analysis (Figure 2(e)) confirmed a significant association of this gene set with cytokine and interferon-related pathways, indicating a tight relationship between TRAIL and interferon signalling in PCa tissue. While XAF1 was significantly overexpressed in our microarray analyses, the expression of XIAP, a functional opponent of XAF1, was not significantly altered in miR-221 overexpressing PC3 cells (Supplementary Material, [Supplementary-material supplementary-material-1]).

### 3.3. PIK3R1 as Direct Target Gene of miR-221 in PCa Cells

**Figure 3:**
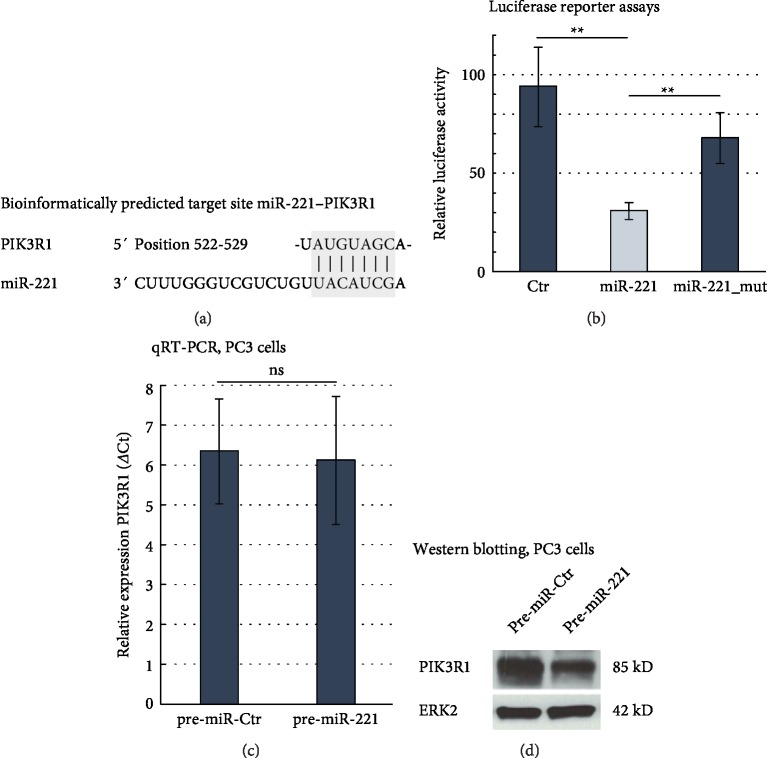
PIK3R1 as a direct target gene of miR-221 in PCa. (a) Bioinformatically predicted miR-221-PIK3R1 binding site. (b) Luciferase reporter assays showing the relative Luciferase activity of pre-miR-Ctr and pre-miR-221 transfected LNCaP cells. The right column shows the luciferase activity for pre-miR-221 transfected LNCaP cells with a mutated PIK3R1 binding site. Data represent mean + SD from five independent experiments, ∗*p* < 0.05, ∗∗*p* < 0.01. (a, b) ANOVA with Bonferroni post hoc testing. (c) qRT-PCR results showed no significant change in PIK3R1 expression depending on miR-221 expression levels in PC3 cells (pre-miR-221 vs. pre-miR-Ctr, 48 h p. t.). Data represent mean + SD from five independent experiments. (d) Western blotting experiments showed a decrease in protein expression of PIK3R1 after restoration of cellular miR-221 expression (PC3 cells, 48 h p. t.). ERK2 was used as housekeeping protein.

To further characterise the role of miR-221 in TRAIL sensitivity, we searched for potential miR-221 target genes involved in TRAIL signalling. As illustrated in Figure 3(a), PIK3R1 is bioinformatically predicted as miR-221 target gene by the *TargetScan* web resource. To verify this targeting *in vitro*, we performed Luciferase reporter assays in LNCaP and PC3 cells (Figure 3(b), Supplementary Material [Supplementary-material supplementary-material-1]) and saw a highly significant decrease in relative Luciferase activity (from 93.9 to 31.3 RLU, *p* < 0.01) in pre-miR-221 co-transfected LNCaP cells compared to control transfections. Mutation of the predicted miR-221 binding site nearly restored the initial luciferase activity in the pre-miR-221 treated LNCaP cells (31.1–68.2 RLU, *p* < 0.01). We analysed the miR-221-mediated regulation of PIK3R1 by qRT-PCR (Figure 3(c)), finding no significant expression change of PIK3R1 on RNA level (6.12 ± 1.61ΔCt in pre-miR-221-transfected cells vs. 6.35 ± 1.31ΔCt in control transfected cells, *p* = 0.85). Instead, Western blotting experiments confirmed a robust reduction of PIK3R1 protein expression in pre-miR-221 transfected PC3 cells compared to control (Figure 3(d)).

### 3.4. Knockdown of SOCS3 and PIK3R1 Significantly Augmented TRAIL Effects in PC3 Cells

**Figure 4:**
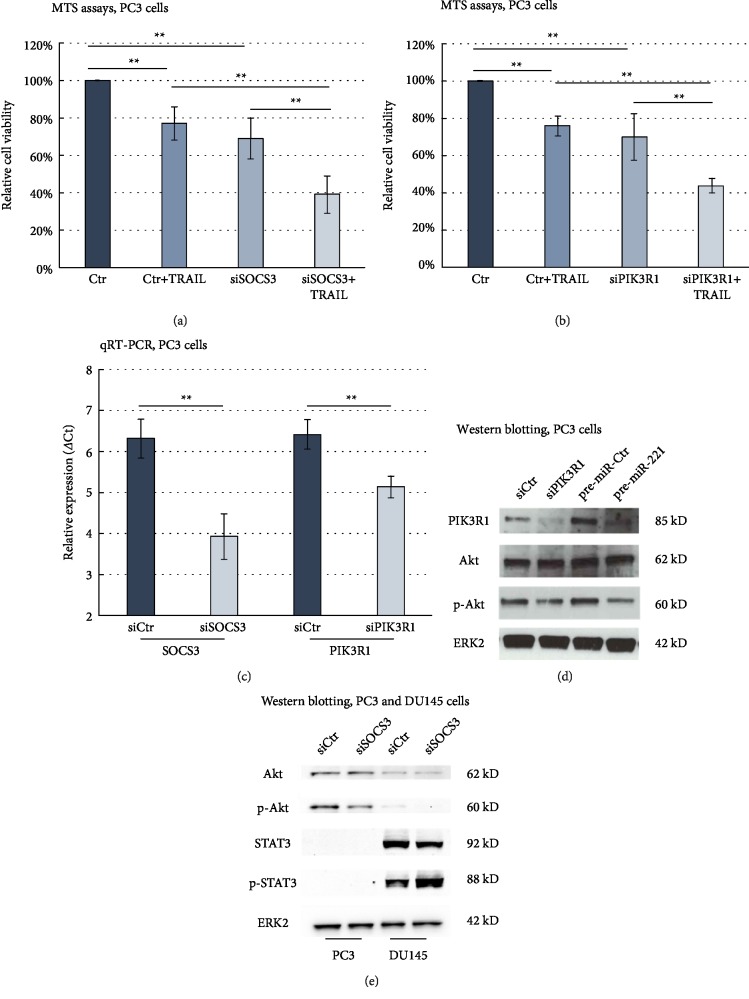
Effects of siRNA-mediated downregulation of SOCS3 and PIK3R1 on TRAIL sensitivity of PC3 cells. (a, b) Downregulation of SOCS3 (a) and PIK3R1 (b) sensitises PC3 cells towards TRAIL-mediated proliferation inhibition. Results shown for PC3 cells 120 h p. t. (a, b) Relative cell viability was normalised to the viability of control cells, which was arbitrarily set as 100%. (c) qRT-PCR results confirmed a significant downregulation of SOCS3 as well as PIK3R1 after siRNA transfection in PC3 cells (48 h p. t.). (a, b, c): Data represent mean + SD from five independent experiments (∗∗*p* < 0.01). (a, b) ANOVA with Bonferroni post hoc testing. (d) Western blotting experiments for PC3 cells showing PIK3R1, Akt, p-Akt and ERK2 protein expression depending on siPIK3R1, pre-miR-221 and control transfections (48 h p. t.). (e) Western blotting experiments in PC3 and DU145 cells showing the expression of Akt, p-Akt, STAT3, p-STAT3 and ERK2 in siCtr- and siSOCS3-transfected cells (48 h p. t.). (a, b, c, d, e) Control cells (Ctr or siCtr) were transfected with scrambled, nontargeting pre-miRNA (pre-miR-Ctr) or siRNA-Ctr (siCtr), respectively.

Next, we examined the effect of the miR-221 target genes SOCS3 and PIK3R1 on TRAIL signalling by performing MTS assays of PCa cells with/without TRAIL administration after siRNA-mediated downregulation of both miR-221 target genes (Figures 4(a) and 4(b)). As illustrated in Figure 4(c), siRNA treatment led to a highly significant decrease in expression for both genes.

With 69.1 ± 10.6% viable cells compared to control (*p* < 0.01), siSOCS3 transfection induced a significant proliferation inhibition in PC3 cells. Moreover, siSOCS3 transfection induced a significant rise in TRAIL sensitivity–from 77 ± 9% viable cells in TRAIL-treated cells after control transfection to 39.4 ± 9.9% viable cells after TRAIL treatment in siSOCS3-transfected PC3 cells (*p* < 0.01).

Regarding PIK3R1 downregulation (Figure 4(b)), PC3 cells also showed a significant decrease in survival after siPIK3R1 transfection compared to control (70.3 ± 12.5%, *p* < 0.01). As seen for siSOCS3 transfection, siRNA-mediated downregulation of PIK3R1 also had a highly significant effect on TRAIL sensitivity (75.5 ± 4.7%–44.3 ± 4% viable cells, *p* < 0.01).

Figures 4(d) and 4(e) shows Western blotting experiments with siPIK3R1, siSOCS3, and pre-miR-221. First, transfection with siPIK3R1 sufficiently reduced the amount of PIK3R1 protein in PC3 cells, indicating an efficient siRNA-mediated downregulation of PIK3R1. Besides, as already shown in Figure 3(d), pre-miR-221 transfection also reduced the protein level of PIK3R1. siRNA-mediated downregulation of PIK3R1 or SOCS3 as well as pre-miR-221 transfection lowered the protein amount of phospho-Akt, while no significant changes of Akt protein expression could be seen in any transfection. These results indicate that the activation of Akt in miR-221 overexpressing cells might be regulated by a miR-221-mediated inhibition of both PIK3R1 and SOCS3. In DU145 cells–with generally very low Akt levels–the phospho-Akt expression nearly vanished after siSOCS3 transfection (Figure 4(e)). Since we previously have observed a strong activation of STAT3 in miR-221 overexpressing DU145 cells, we finally analysed the role of SOCS3 in STAT3 activation by miR-221. Figure 4(e) shows that siRNA-mediated knockdown of SOCS3 reduced phospho-STAT3 expression levels–whereas STAT3 expression remained unchanged. Based on these results, we concluded that STAT3 activation in miR-221 overexpressing PCa cells was at least partially caused by miR-221-mediated SOCS3 inhibition.

## 4. Discussion

miR-221 is overexpressed in many malignancies including colorectal and pancreatic cancer [20, 21]. One reason for the *oncogenic branding* across various cancer entities was the miR-221-mediated resistance towards the cytokine TRAIL [6]. However, miR-221 is downregulated in malignancies such as Gastrointestinal Stromal Tumours (GIST) [22] and NSCLC [23] and a meta-analysis of several independent studies demonstrated a strong and frequent downregulation of miR-221 in PCa tissue [10]. Moreover, our study group described downregulation of miR-221 in high risk PCa [9] and showed that miR-221 expression levels in prostatectomy specimen had a significant prognostic impact on overall and cancer-specific survival in two independent high risk PCa cohorts [11]. This led us to take a closer look at the potential interaction of miR-221 and TRAIL in PCa cells.

### 4.1. A Novel Role for miR-221 in TRAIL Signalling

Instead of inducing TRAIL resistance as shown in other malignancies, miR-221 played the opposite role in our setting: restoring miR-221 expression sensitized PC3 and DU145 cells towards TRAIL in terms of decreased viability and in terms of stronger induction of apoptosis. This novel trait of miR-221 seemed to fit well with the miR-221-mediated augmentation of interferon signalling in PCa demonstrated by our group [11], as interferon and TRAIL signalling were shown to act synergistically [12, 13]. Of note, this close link to interferon could be one reason, why LNCaP cells could not be sensitized towards TRAIL by higher miR-221 expression levels , as this cell line is reported to be interferon unresponsive [11, 24].

Apart from reported TRAIL/interferon synergisms in other malignancies, we examined a potential interaction of these pathways in PCa *in silico. *We found that genes strongly co-expressed with TNFSF10 in PCa tissue also were frequently and significantly associated with JAK-STAT-mediated interferon signalling. Depending on this observation, we decided to further analyse the molecular changes leading to the modified TRAIL sensitivity in miR-221 overexpressing PCa cells.

### 4.2. The miR-221 Target Gene SOCS3 as Inhibitor of TRAIL Signalling in PCa Cells

Our study group demonstrated that SOCS3 is a target gene of miR-221 and that its downregulation provoked a broad gene signature of interferon-induced genes [11]. We now confirmed the miR-221-mediated augmentation of interferon signalling by *DAVID Functional Annotation* analyses of microarray data showing immune-related and specifically interferon-associated pathways significantly overrepresented in miR-221 overexpressing PC3 cells. Within these microarray experiments, we searched for genes involved in interferon and TRAIL signalling and found TNFSF10, XAF1, and STAT1 among the most upregulated genes. Noteworthy, restoration of cellular miR-221, causing a reduced SOCS3 expression, strengthened endogenous TRAIL expression in PC3 cells by significantly inducing the TRAIL encoding gene TNFSF10. Additionally, XAF1 is regarded as a crucial regulator of TRAIL sensitivity by linking interferon and TRAIL signalling [25] and by neutralizing TRAIL resistance induced by its functional competitor XIAP (X-linked inhibitor of apoptosis). This anti-apoptotic factor works by directly blocking the caspase cascade and preventing subsequent cell death [26, 27]. Further analysis of XAF1 together with XIAP expression within our microarray experiments revealed stable levels of XIAP accompanied by a strong induction of XAF1 expression.

In concordance with our results, it was also shown that SOCS3 counteracts TRAIL effects in PCa cells by directly binding DR 4 (death receptor 4), thereby inhibiting the downstream apoptotic cascade [28]. Additionally, we examined the effect of siRNA-mediated downregulation of SOCS3 on TRAIL sensitivity and observed that downregulation of SOCS3 significantly sensitized PC3 cells towards a TRAIL-induced reduction in cell viability, thereby mimicking the effect of high miR-221 expression levels. Our results indicate that miR-221 effects on TRAIL sensitivity are at least partly caused by miR-221 targeting SOCS3.

We previously detected phospho-Akt downregulation and STAT3 phosphorylation in miR-221 overexpressing cells. Here, we show decreased Akt activation and increased STAT3 activation in SOCS3-downregulated PCa cells—thereby indicating a critical role of SOCS3 for miR-221 function in PCa cells. Nevertheless, an anti-tumorigenic role of phospho-STAT3 is conflicting, because of its reported oncogenic function. However, STAT3 activation can induce apoptosis under certain conditions in various cancer types including PCa. Moreover, SOCS3 downregulation determined reduced proliferation rates and an increased apoptotic response by converting the anti-apoptotic STAT3 function into pro-apoptotic [29]. In addition, it was shown that reduced SOCS3 expression enhanced the IFN*γ* responsiveness, indicating a regulation of IFN*γ* sensitivity in PCa cells by SOCS3 [11]. Therefore, we suggest that the regulation of both Akt and STAT3 activation by SOCS3 might explain at least partially the anti-proliferative and pro-apoptotic activity of miR-221 and the modulation of sensitivity against TRAIL-mediated apoptosis in PCa cells.

Regarding the contradictory effects of miR-221 in LNCaP cells, it was shown that SOCS3 expression levels were negligible in Androgen-dependent LNCaP cells compared to markedly higher SOCS3 levels in the androgen-independent cell lines PC3 and DU145 [30]. This observation might at least partially explain the differing effects of TRAIL and miR-221 overexpression in LNCaP cells, thereby highlighting the importance of the molecular microenvironment for the function of miR-221 in PCa.

### 4.3. PIK3R1 as Target Gene of miR-221 and Its Role as Inhibitor of TRAIL Signalling in PCa Cells

We further looked for additional miR-221 target genes with a putative role in TRAIL signalling and identified PIK3R1 as an interesting candidate. This gene is reported to be regulated by miR-221 in endothelial cells during embryogenesis [31] and has a miR-221 binding site in its 3′ UTR. Luciferase reporter assays and Western blotting experiments confirmed a miR-221 targeting of PIK3R1. We then examined the effect of siRNA-mediated downregulation of PIK3R1 on TRAIL sensitivity in PC3 cells. Downregulation of PIK3R1 caused a significant reduction in cell viability and significantly sensitised PC3 cells towards TRAIL, thereby mimicking the effects of a miR-221 restoration. In addition, a study reported PIK3R1 to counteract TRAIL-induced apoptosis in colorectal cancer cells [32], confirming a role of PIK3R1 in the regulation of TRAIL sensitivity also in other cancer entities.

In general, PIK3R1 represents an interesting miR-221 target gene in PCa: On the one hand, PIK3R1 plays a crucial role within the PI3K/Akt pathway, which is regarded as a key driver of PCa progression [33]. On the other hand, PI3K/Akt is a key factor for TRAIL resistance in many malignancies including PCa [34–37]. However, PIK3R1 usually is regarded as a tumour suppressor by serving as the regulatory subunit of PI3K [38]. Supported by the notion of relatively frequent PIK3R1 mutations in PCa, this tumour suppressor role has also been propagated in this entity [39, 40]. Instead, as shown by our cell viability assays, downregulation of PIK3R1 not only reduced viability but augmented TRAIL effects in PC3 cells. This oncogenic function of PIK3R1 in our setting is supported by Western blotting experiments demonstrating lower amounts of phospho-Akt after siRNA-mediated downregulation of PIK3R1 and after restoration of miR-221 expression in PC3 cells. These findings seem to be in line with recent publications claiming an oncogenic role for PIK3R1 in several malignancies such as Glioblastoma multiforme and Hepatocellular carcinoma by fostering PI3K/Akt activation [41–43].

In conclusion, we found two independent miR-221 target genes, SOCS3 and PIK3R1, both critically involved in the regulation of TRAIL sensitivity in PCa cells. Based on the observed and described functions of SOCS3 and PIK3R1, we suggest that the role of miR-221 in TRAIL signalling is strongly mediated by both genes. For SOCS3, TRAIL inhibiting effects seem to be caused by a direct binding to DR4 [28] and by counteracting a TRAIL-friendly interferon signature consisting of crucial mediators such as TNFSF10 and XAF1. Given the overexpression of XAF1 after miR-221 restoration in PC3 cells and the interaction with its anti-apoptotic counterpart XIAP [26], it seems interesting that several studies reported a tight positive interrelationship between XIAP and Akt [44, 45]. Therefore, regulation of the PI3K/Akt pathway by both, SOCS3 and PIK3R1 might be crucial for the TRAIL sensitising function of miR-221 in PCa cells.

Noteworthy, apart from the functional aspects of TRAIL sensitization by miR-221 shown here *in vitro*, more research is needed to elucidate the role and the significance of miR-221 targeting PIK3R1 and SOCS3 in PCa *in vivo*.

## 5. Conclusions

Instead of inducing TRAIL resistance as previously demonstrated in other malignancies, miR-221 sensitised PC3 and DU145 cells towards TRAIL effects. After having shown that miR-221 strengthens interferon signalling by regulating SOCS3 [11], augmentation of TRAIL effects constitutes a second tumour suppressive role in PCa. Functionally, miR-221 augmented TRAIL signalling by targeting SOCS3 and PIK3R1 in PCa cells. Especially the role and the extent of the PIK3R1 regulation will need further examination, as PIK3R1 has been shown to serve as the regulatory subunit of PI3K. However, proliferation inhibition after siRNA-mediated downregulation of PIK3R1 as well as sensitization towards the effects of TRAIL in PC3 cells demonstrated an oncogenic function of PIK3R1 *in vitro*. Given the prognostic potential of a miR-221 downregulation in PCa, our results suggest, that high risk stages in PCa could be linked to enhanced TRAIL resistance. Moreover, miR-221 in combination with SOCS3 and PIK3R1 expression could serve as a potential predictive biomarker model for TRAIL response in aggressive PCa.

## Data Availability

Previously reported microarray data were used to support this study and are accessible through GEO Series accession number GSE45627 (deposited in NCBI Gene Expression Omnibus). These prior studies (and datasets) are cited at relevant places within the text as references [11].

## Supplementary Materials

Supplementary Materials Expression of XAF1 and XIAP in PC3 cells depending on pre-miR-221 transfection.

## References

[1] Gura T. (1997). How TRAIL kills cancer cells, but not normal cells. *Science*.

[2] Stuckey D. W., Shah K. (2013). TRAIL on trial: preclinical advances in cancer therapy. *Trends in Molecular Medicine*.

[3] Hellwig C. T., Rehm M. (2012). TRAIL signaling and synergy mechanisms used in TRAIL-based combination therapies. *Molecular Cancer Therapeutics*.

[4] Lemke J., Von Karstedt S., Zinngrebe J., Walczak H. (2014). Getting TRAIL back on track for cancer therapy. *Cell Death & Differentiation*.

[5] Lu T., Shao N., Ji C. (2013). Targeting microRNAs to modulate TRAIL-induced apoptosis of cancer cells. *Cancer Gene Therapy*.

[6] Garofalo M., Di Leva G., Romano G. (2009). miR-221&222 regulate TRAIL resistance and enhance tumorigenicity through PTEN and TIMP3 downregulation. *Cancer Cell*.

[7] Lu Q., Lu C., Zhou G.-P., Zhang W., Xiao H., Wang X.-R. (2010). MicroRNA-221 silencing predisposed human bladder cancer cells to undergo apoptosis induced by TRAIL. *Urologic Oncology: Seminars and Original Investigations*.

[8] Galardi S., Mercatelli N., Farace M. G., Ciafre S. A. (2011). NF-kB and c-Jun induce the expression of the oncogenic miR-221 and miR-222 in prostate carcinoma and glioblastoma cells. *Nucleic Acids Research*.

[9] Spahn M., Kneitz S., Scholz C. J. (2010). Expression of microRNA-221 is progressively reduced in aggressive prostate cancer and metastasis and predicts clinical recurrence. *International Journal of Cancer*.

[10] Yang J., Zhang J.-Y., Chen J., Xu Y., Song N.-H., Yin C.-J. (2014). Prognostic role of microRNA-221 in various human malignant neoplasms: a meta-analysis of 20 related studies. *PLoS ONE*.

[11] Kneitz B., Krebs M., Kalogirou C. (2014). Survival in patients with high-risk prostate cancer is predicted by miR-221, which regulates proliferation, apoptosis, and invasion of prostate cancer cells by inhibiting IRF2 and SOCS3. *Cancer Research*.

[12] Clarke N., Jimenez-Lara A. M., Voltz E., Gronemeyer H. (2004). Tumor suppressor IRF-1 mediates retinoid and interferon anticancer signaling to death ligand TRAIL. *The EMBO Journal*.

[13] Miura Y., Tsujioka T., Nishimura Y. (2006). TRAIL expression up-regulated by interferon-gamma via phosphorylation of STAT1 induces myeloma cell death. *Anticancer Research*.

[14] Chandrashekar D. S., Bashel B., Balasubramanya S. A. H. (2017). UALCAN: a portal for facilitating tumor subgroup gene expression and survival analyses. *Neoplasia*.

[15] Fabregat A., Jupe S., Matthews L. (2018). The reactome pathway knowledgebase. *Nucleic Acids Research*.

[16] Fabregat A., Sidiropoulos K., Viteri G. (2017). Reactome pathway analysis: a high-performance in-memory approach. *BMC Bioinformatics*.

[17] Huang D. W., Sherman B. T., Lempicki R. A. (2008). Systematic and integrative analysis of large gene lists using DAVID bioinformatics resources. *Nature Protocols*.

[18] Huang D. W., Sherman B. T., Lempicki R. A. (2008). Bioinformatics enrichment tools: paths toward the comprehensive functional analysis of large gene lists. *Nucleic Acids Research*.

[19] Agarwal V., Bell G. W., Nam J.-W., Bartel D. P. (2015). Predicting effective microRNA target sites in mammalian mRNAs. *eLife*.

[20] Lee E. J., Gusev Y., Jiang J. (2007). Expression profiling identifies microRNA signature in pancreatic cancer. *International Journal of Cancer*.

[21] Sun K., Wang W., Zeng J.-J., Wu C.-T., Lei S.-T., Li G.-X. (2011). MicroRNA-221 inhibits CDKN1C/p57 expression in human colorectal carcinoma. *Acta Pharmacologica Sinica*.

[22] Koelz M., Lense J., Wrba F., Scheffler M., Dienes H. P., Odenthal M. (2011). Down-regulation of miR-221 and miR-222 correlates with pronounced Kit expression in gastrointestinal stromal tumors. *International Journal of Oncology*.

[23] Yu S.-L., Chen H.-Y., Chang G.-C. (2008). MicroRNA signature predicts survival and relapse in lung cancer. *Cancer Cell*.

[24] Dunn G. P., Sheehan K. C., Old L. J., Schreiber R. D. (2005). IFN unresponsiveness in LNCaP cells due to the lack of JAK1 gene expression. *Cancer Research*.

[25] Leaman D. W., Chawla-Sarkar M., Vyas K. (2002). Identification of X-linked inhibitor of apoptosis-associated factor-1 as an interferon-stimulated gene that augments TRAIL Apo2L-induced apoptosis. *Journal of Biological Chemistry*.

[26] Liston P., Fong W. G., Kelly N. L. (2001). Identification of XAF1 as an antagonist of XIAP anti-Caspase activity. *Nature Cell Biology*.

[27] Shibata T., Noguchi T., Takeno S., Gabbert H. E., Ramp U., Kawahara K. (2008). Disturbed XIAP and XAF1 expression balance is an independent prognostic factor in gastric adenocarcinomas. *Annals of Surgical Oncology*.

[28] Horndasch M., Culig Z. (2011). SOCS-3 antagonizes pro-apoptotic effects of TRAIL and resveratrol in prostate cancer cells. *The Prostate*.

[29] Lu Y., Fukuyama S., Yoshida R. (2006). Loss of SOCS3 gene expression converts STAT3 function from anti-apoptotic to pro-apoptotic. *Journal of Biological Chemistry*.

[30] Handle F., Erb H. H., Luef B. (2016). SOCS3 modulates the response to enzalutamide and is regulated by androgen receptor signaling and CpG methylation in prostate cancer cells. *Molecular Cancer Research*.

[31] Nicoli S., Knyphausen C.-P., Zhu L. J., Lakshmanan A., Lawson N. D. (2012). miR-221 is required for endothelial tip cell behaviors during vascular development. *Developmental cell*.

[32] Rychahou P. G., Murillo C. A., Evers B. M. (2005). Targeted RNA interference of PI3K pathway components sensitizes colon cancer cells to TNF-related apoptosis-inducing ligand (TRAIL). *Surgery*.

[33] Bitting R. L., Armstrong A. J. (2013). Targeting the PI3K/Akt/mTOR pathway in castration-resistant prostate cancer. *Endocrine-Related Cancer*.

[34] Chen X., Thakkar H., Tyan F. (2001). Constitutively active Akt is an important regulator of TRAIL sensitivity in prostate cancer. *Oncogene*.

[35] Dieterle A., Orth R., Daubrawa M. (2009). The Akt inhibitor triciribine sensitizes prostate carcinoma cells to TRAIL-induced apoptosis. *International Journal of Cancer*.

[36] Opel D., Naumann I., Schneider M., Bertele D., Debatin K.-M., Fulda S. (2011). Targeting aberrant PI3K/Akt activation by PI103 restores sensitivity to TRAIL-induced apoptosis in neuroblastoma. *Clinical Cancer Research*.

[37] Xu J., Zhou J.-Y., Wei W.-Z., Wu G. S. (2010). Activation of the Akt survival pathway contributes to TRAIL resistance in cancer cells. *PloS ONE*.

[38] Taniguchi C. M., Winnay J., Kondo T. (2010). The phosphoinositide 3-kinase regulatory subunit p85α can exert tumor suppressor properties through negative regulation of growth factor signaling. *Cancer Research*.

[39] Gao D., Vela I., Sboner A. (2014). Organoid cultures derived from patients with advanced prostate cancer. *Cell*.

[40] Taylor B. S., Schultz N., Hieronymus H. (2010). Integrative genomic profiling of human prostate cancer. *Cancer Cell*.

[41] Huang C.-Y., Huang X.-P., Zhu J.-Y. (2015). miR-128-3p suppresses hepatocellular carcinoma proliferation by regulating PIK3R1 and is correlated with the prognosis of HCC patients. *Oncology Reports*.

[42] Huang X. P., Hou J., Shen X. Y. (2015). MicroRNA-486-5p, which is downregulated in hepatocellular carcinoma, suppresses tumor growth by targeting PIK3R1. *The FEBS journal*.

[43] Weber G. L., Parat M.-O., Binder Z. A., Gallia G. L., Riggins G. J. (2011). Abrogation of PIK3CA or PIK3R1 reduces proliferation, migration, and invasion in glioblastoma multiforme cells. *Oncotarget*.

[44] Asselin E., Mills G. B., Tsang B. K. (2001). XIAP regulates Akt activity and caspase-3-dependent cleavage during cisplatin-induced apoptosis in human ovarian epithelial cancer cells. *Cancer Research*.

[45] Gagnon V., Van Themsche C., Turner S., Leblanc V., Asselin E. (2008). Akt and XIAP regulate the sensitivity of human uterine cancer cells to cisplatin, doxorubicin and taxol. *Apoptosis*.

