# Supplementary material for: miR-221 Augments TRAIL-Mediated Apoptosis in Prostate Cancer Cells by Inducing Endogenous TRAIL Expression and Targeting the Functional Repressors SOCS3 and PIK3R1
Source: Biomed Res Int. 2019 Nov 14;2019:6392748. doi: 10.1155/2019/6392748 (PMC6881584; doi:10.1155/2019/6392748)
Supplement: Supplementary Materials — Expression of XAF1 and XIAP in PC3 cells depending on pre-miR-221 transfection. [file 6392748.f1.pdf]

Supplementary data

Expression of XAF1 and XIAP in PC3 cells depending on pre-miR-221 transfection

probeset ID	GENENAME	SYMBOL	Nr 1_Pre-Mir-221	Nr 1_pre 221	Nr 3_Ctrl	logFC
235222_x_at	X-linked inhibitor of apoptosis	XIAP	24,581	25,601	25,709	-0,135
206536_s_at	X-linked inhibitor of apoptosis	XIAP	22,663	22,817	22,153	-0,535
243026_x_at	X-linked inhibitor of apoptosis	XIAP	23,925	24,820	24,891	-0,136
206537_at	X-linked inhibitor of apoptosis	XIAP	21,646	21,820	21,363	-0,416
225859_at	X-linked inhibitor of apoptosis	XIAP	25,365	24,847	24,955	-0,022
228363_at	X-linked inhibitor of apoptosis	XIAP	26,652	26,483	26,719	-0,053
225858_s_at	X-linked inhibitor of apoptosis	XIAP	25,944	25,728	25,862	-0,010
228617_at	XIAP associated factor 1	XAF1	25,023	28,796	21,684	-4,728
206133_at	XIAP associated factor 1	XAF1	21,958	24,858	19,815	-3,368
242234_at	XIAP associated factor 1	XAF1	21,092	23,388	20,138	-1,769

Table S1. Transient transfection with pre-miR-221 significantly induced the expression of XAF1 compared to control transfections in PC3 cells (48h p. t.) within our microarray experiments. In contrast, no significant change in expression could be detected for XIAP.

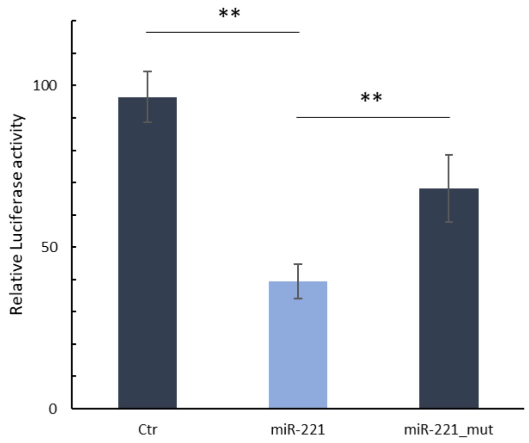

Figure S1. Luciferase reporter assays confirmed a specific binding of miR-221 and PIK3R1 3´UTR in PC3 cells. Compared to pre-miR-Ctr transfected cells, pre-miR-221 transfected PC3 cells displayed a highly significant reduction of relative Luciferase activity. The right column represents the relative Luciferase activity for pre-miR-221 transfected PC3 cells with a mutated PIK3R1 binding site. Data represent mean + SD of four independent experiments. *: p<0.05, **: p<0.01.
